# Targeted Gastrointestinal Delivery of mRNA Lipid Nanoparticles Enables Systemic Vaccination and Glucagon-like Peptide‑1 Metabolic Therapy

**DOI:** 10.1021/acsnano.5c18549

**Published:** 2026-09-28

**Authors:** Abdulraouf M. Abbas, Ramy Ghanim, Sebastian Rudden, David Schultz, Bora Jang, Avraham Shakked, Hyejin Kim, James E. Dahlman, Alex Abramson

**Affiliations:** † Wallace H. Coulter Department of Biomedical Engineering, Emory University School of Medicine and Georgia Institute of Technology, Atlanta, Georgia 30332, United States; ‡ Emory Global Diabetes Research Center of the Woodruff Health Sciences Center and Emory University, Atlanta, Georgia 30322, United States; § School of Chemical and Biomolecular Engineering, 1372 Georgia Institute of Technology, Atlanta, Georgia 30332, United States; ∥ Wearable Intelligent Systems and Healthcare Center, Institute for Matters and Systems, Georgia Institute of Technology, Atlanta, Georgia 30332, United States; ⊥ Division of Digestive Diseases, Emory University School of Medicine, Atlanta, Georgia 30332, United States

**Keywords:** Lipid nanoparticles, mRNA delivery, Gastrointestinal delivery, Pancreatic delivery, GLP-1 mRNA, Vaccine, Oral delivery

## Abstract

Lipid nanoparticles (LNPs) administered parenterally often show poor localization to multiple tissue types, including the pancreas and gastrointestinal (GI) tract. Furthermore, patients typically prefer orally administered drugs to those that are given parenterally. We therefore investigated whether GI LNP administration to buccal, gastric, small intestinal, colonic, and rectal submucosal tissues could simultaneously circumvent parenteral administration while enhancing LNP delivery to previously inaccessible locations. We found that, in addition to local targeting, gastric LNP administration achieved an order of magnitude greater pancreas delivery in rodents than intravenous administration. Gastric dosing of glucagon-like peptide-1 mRNA also improved blood glucose regulation in mice compared with phosphate-buffered saline controls. Additionally, we found that buccal LNP administration targeted local and lymphatic tissues. Furthermore, dosing mice with ovalbumin mRNA resulted in equivalent immunoglobulin G blood titers to those of intramuscular delivery. These results suggest that GI administration can reprogram LNP biodistribution, expanding therapeutic opportunities for both local and systemic nucleic acid delivery.

## Graphical abstract

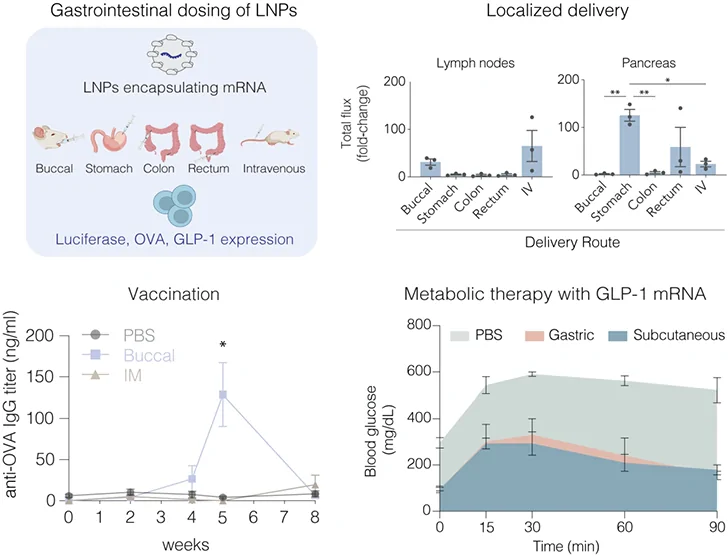

## Introduction

Therapeutic nucleic acids feature in over 20 U.S. Food and Drug Administration-approved products.^1–3^ However, messenger RNA (mRNA) drug products have been limited to intramuscularly administered vaccines, and short interfering RNA (siRNA) products have been limited to intravenous delivery for liver diseases. Nonliver delivery remains challenging, yet nucleic acids show promise in treating preclinical models of gastrointestinal diseases, including gastroesophageal cancer, gastric carcinoma, and ulcerative colitis, as well as pancreatic diseases such as cancer and diabetes.^4–11^ To improve nonliver delivery, laboratories alter lipid nanoparticle (LNP) chemistry by adding active targeting ligands or permanently charged helper lipids.^38–15^ An alternative strategy involves altering the administration route. Scientists have reported improved lung delivery using nebulization,^16^ improved lymphatic delivery using subcutaneous or intramuscular administration,^17,18^ and improved pancreas delivery via intraperitoneal administration^19^ yet it remains unclear whether other delivery routes alter LNP tropism.

Given the advent of new technologies for the gastrointestinal (GI) delivery of biologics^20–24^ and evidence that patients prefer oral alternatives to parenteral administration,^25,26^ we found this question compelling. Specifically, scientists have reported approaches to avoid the harsh environment and diffusional barriers of the GI tract,^27^ including endoscopic injections,^23^ miniaturized ingestible microneedle pills^21^ and patches,^28^ jet injection pills,^4,24^ ultrasound mediated enemas,^29^ and novel formulations.^30–32^ All of these have generated data consistent with local endoluminal and buccal nucleic acid uptake. However, it remains unclear how altering the GI-related route of administration affects delivery systemically. The on- and off-target delivery ratios as a function of administration route are especially important given that unwanted liver delivery is an established challenge in the field of nucleic acid therapeutics.

Here, we systematically investigated LNP-mRNA delivery to the submucosa of clinically relevant GI organs, including buccal, gastric, small intestine, colonic, and rectal tissues. We found that the site of administration dramatically alters the biodistribution of translated proteins, unlocking opportunities for both local and systemic targets. For example, after submucosal buccal LNP injections, we observed noticeable delivery to local buccal tissue as well as inguinal lymph nodes. Furthermore, delivering an ovalbumin mRNA cargo to the buccal regions resulted in systemic immunoglobulin G (IgG) blood serum titers. Buccal delivery matched intramuscular vaccination IgG titers at equivalent 1 μg doses and retained measurable systemic IgG responses at a 10-fold lower dose, where intramuscular administration failed to generate an immune response. Separately, we discovered that gastric delivery resulted in significantly higher uptake locally as well as in the pancreas compared to intravenous administration. This delivery improvement was coupled with significantly lower uptake in other organs such as the liver, lungs, heart, and spleen. Combining these administration routes with formulation modifications, such as adding a cationic helper lipid, further improved pancreas targeting. To demonstrate the metabolic therapeutic potential of gastric nucleic acid administration, we delivered a glucagon-like peptide-1 (GLP-1) mRNA cargo injection to the gastric submucosa and noted that it resulted in improved blood glucose control in mice compared to controls. Our studies highlight that optimizing GI delivery routes and LNP composition may lead to differentiated LNP tropism as well as provide meaningful therapeutic potential *in vivo.*

## Results

### Gastrointestinal LNP Administration Improves mRNA Delivery Locally and in the Pancreas While Decreasing mRNA Delivery to the Liver, Lungs, and Spleen

We first measured LNP trafficking and delivery as a function of the GI-related route of administration. We formulated an LNP using the clinically validated ionizable lipid SM-102 and the zwitterionic helper lipid 1,2-distearoyl-*sn*-glycero-3-phosphocholine (DSPC) (termed LNP^neut^) with mRNA encoding anchored nanoluciferase (aNLuc).^33,34^ Then, we injected this formulation into C57BL/6 mice via the buccal, gastric, colonic, and rectal submucosal tissues at a dose of 1 mg kg^–1^ (Figure 1a). As a control, we administered the LNPs intravenously to mice. Separately, we injected these LNPs into the small intestine of Sprague–Dawley rats, since small intestine injections in mice were not feasible (Figure S1). LNP^neut^ had a hydrodynamic diameter equal to 78 nm, a 98% encapsulation efficiency, and a polydispersity index (PDI) < 0.3 (Figure 1b).

**Figure 1:**
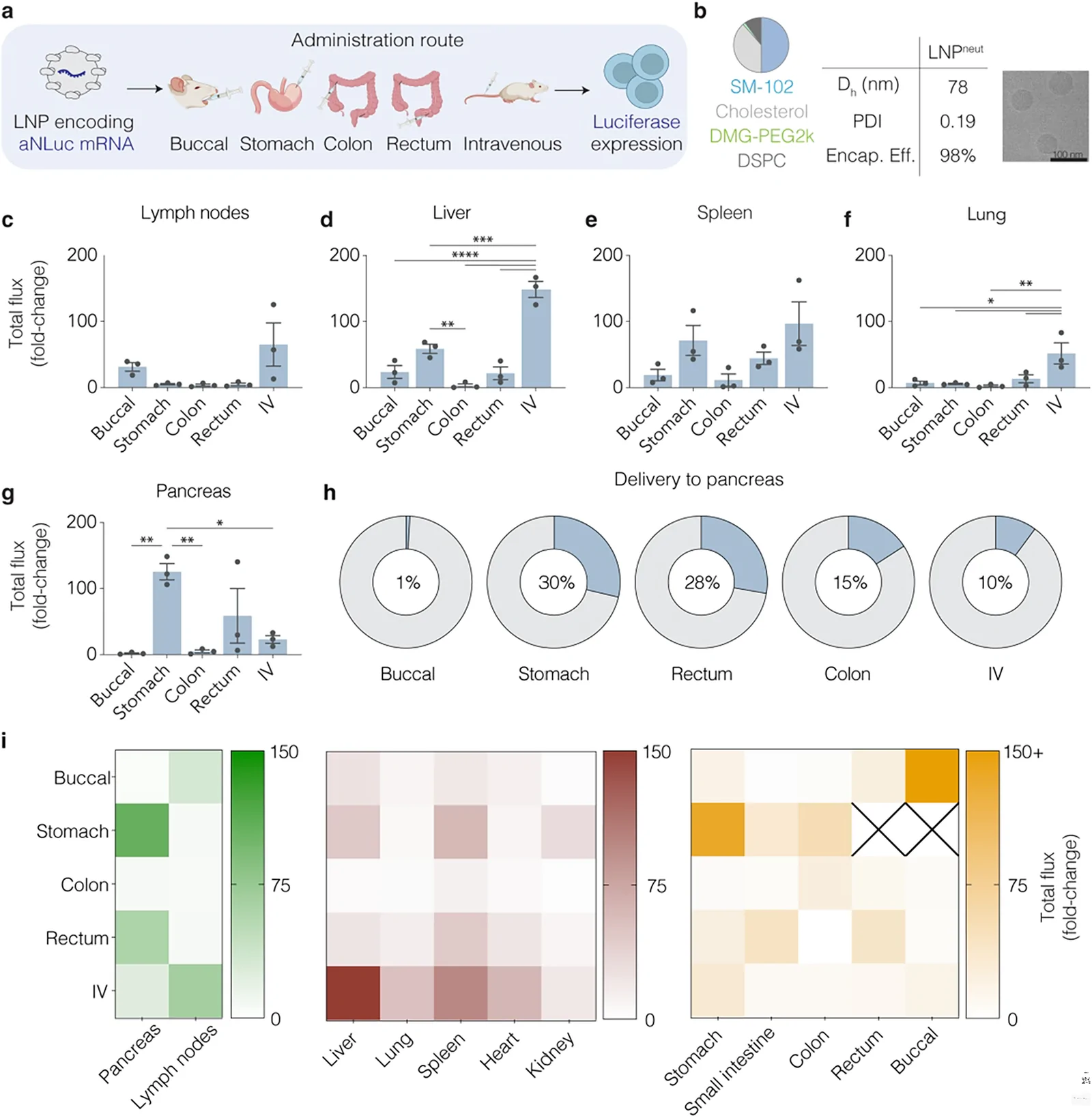
*In vivo* gastrointestinal delivery of aNLuc mRNA with LNP^neut^ revealed pancreas-targeting upon stomach injection. (a) aNLuc mRNA was administered at 1 mg kg^–1^ intravenously (IV) or into the buccal, colon, rectum, or stomach tissue. IVIS imaging at 18 h following dosing revealed luciferase expression. (b) Formulation details of LNP^neut^ and physicochemical characteristics: Hydrodynamic diameter (*D*_h_), polydispersity index (PDI), encapsulation efficiency, and representative cryogenic transmission electron microscope image. DMG-PEG2k is 1,2-dimyristoyl-rac-glycero-3-methoxypolyethylene glycol. DSPC is 1,2-distearoyl-*sn*-glycero-3-phosphocholine. Total flux fold-change relative to phosphate-buffered saline (PBS) treated group of (c) lymph nodes, (d) liver, (e) spleen, (f) lung, and (g) pancreas. (h) Percentage of pancreas total flux expression out of 10 organs from the organ screen [Figure S3]. (i) Heatmap showing total flux (fold-change) across administration routes of pancreas and lymph nodes (green), off-target organs (red), and gastrointestinal (GI) organs (orange). Crossed-out cells indicate organs that were not collected (*n* = 3 mice. Individual data points, mean ± SEM. One-Way ANOVA with Tukey’s correction. *p < 0.05; **p < 0.01; ***p < 0.001; ****p < 0.0001).

Eighteen hours after injection, we collected the pancreas, spleen, kidney, liver, lung, heart, lymph nodes, buccal tissue, small intestine, colon, stomach, and rectum. We then quantified luciferase activity via an *in vivo* imaging system (IVIS). We calculated the total flux fold change for each organ by dividing the total flux (p s^–1^) of an organ from an LNP-treated animal by the average phosphate-buffered saline (PBS)-treated counterpart. Buccal injections resulted in noticeable lymph node delivery (Figure 1c) despite the majority of organ-wide expression occurring locally in the buccal tissue (Figure S3a). The stomach injections resulted in the greatest pancreas absolute luminescence fold change compared to other routes of administration (Figure 1g) and the highest percentage of delivery to the pancreas (Figure 1h). Also of note, GI organ administration resulted in local uptake at the site of injection, delivering to GI organs not traditionally targeted during intravenous administration (Figures S2 and S3). By contrast, liver, lung, spleen, and heart luminescence were highest in the intravenous group (Figures 1d–f and S3). To control for the possibility that LNPs delivered to stomach tissue were leaking out of the stomach into the intraperitoneal space, we injected methylene blue into the stomach and palpated 50 times with a cotton gauze. We observed no leakage (Figure S4). Utilizing this screen, we further explored the impact of lymphatic targeting on vaccine delivery and pancreas targeting on metabolic therapeutic delivery.

### Ovalbumin mRNA LNP Administration via Buccal and Stomach Tissues Generates Systemic IgG Titers

We evaluated the gastrointestinal submucosa as a viable route for mRNA vaccine delivery. First, we formulated LNP^neut^ with either 0.1 μg or 1 μg of ovalbumin (OVA) mRNA. Then, we administered these doses to mice via a buccal, intragastric, or intramuscular (IM) injection on day 0 (prime) and day 21 (boost). Following the injections, we performed a longitudinal analysis of systemic anti-OVA IgG titers using an enzyme-linked immunosorbent assay (ELISA) (Figure 2a).

**Figure 2:**
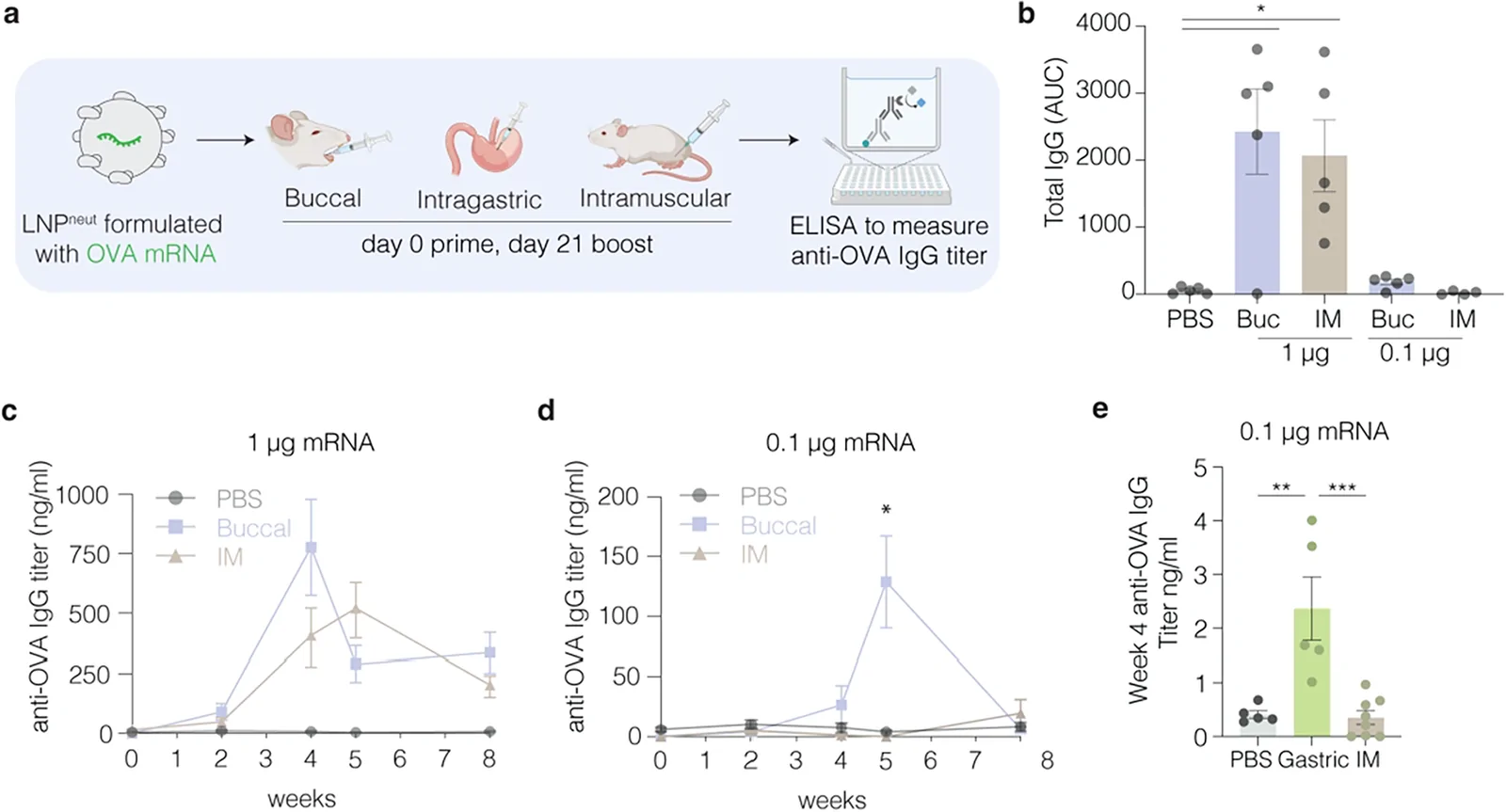
*In vivo* gastrointestinal delivery of ovalbumin (OVA) mRNA with LNP^neut^ enables systemic humoral response. (a) Ovalbumin mRNA was administered at 1 μg buccal (Buc) and intramuscular (IM) and 0.1 μg (gastric, buccal, and intramuscular) at week 0 (prime) and week 3 (boost). Blood serum was processed and analyzed for anti-OVA immunoglobulin G (IgG) antibodies. They were compared to phosphate-buffered saline (PBS) dosed controls. (b) Week 4 ovalbumin-specific serum antibodies after 2 doses of 0.1 or 1 μg mRNA via Buccal and intramuscular (IM) routes. AUC, area under the curve. (c, d) Time course of antibody generation and circulation after (c) 1 μg and (d) 0.1 μg doses at weeks 0 and 3 via buccal and IM routes. (e) Week 4 ovalbumin-specific serum antibodies after 2 doses of 0.1 μg mRNA at weeks 0 and 3 via the Gastric and IM routes. Individual data points, Mean ± SEM. *N* = 5 mice. Multiple unpaired *t* tests with FDR correction (two-stage step-up method; Benjamini, Krieger, and Yekutieli; *Q* = 1%). *p < 0.05; **p < 0.01; and ***p < 0.001.

At the 1 μg dose, both buccal and IM ovalbumin mRNA delivery produced comparable cumulative IgG responses over 8 weeks (Figure 2b,c). Notably, buccal delivery at the 0.1 μg dose still elicited detectable systemic IgG responses at weeks 4 and 5, whereas the IM cohort of mice at this dose remained indistinguishable from PBS controls throughout the study (Figure 2d). At the 0.1 μg dose, mice receiving intragastric administration exhibited significantly elevated week-4 anti-OVA IgG titers compared to both PBS controls and IM-dosed animals (Figure 2e), though absolute titer levels were modest relative to the buccal cohorts. During a dose escalation study, 1 μg of intragastric doses was limited by poor therapeutic tolerance that led to increased mortality rates (Methods), but buccally dosed animals tolerated both doses. These data demonstrate that submucosal GI administration of LNP-formulated mRNA can elicit systemic humoral immunity and provide a potential alternative to conventional intramuscular vaccination.

### LNP Chemistry and Gastric Route of Administration Improve Pancreas Delivery

We evaluated whether formulating an LNP with a charged helper lipid improved pancreas targeting during gastric delivery (Figure 3a). To control for the behavior of other LNP components, we simply replaced the zwitterionic DSPC with cationic dimethyldioctadecylammonium (DDAB); we named this LNP^cat^. Similar to LNP^neut^, LNP^cat^ formed LNPs with a 96 nm hydrodynamic diameter, 98% encapsulation efficiency, and a PDI < 0.3 (Figure 3b). Additionally, while the formulation of an anionic LNP counterpart was explored, substituting the zwitterionic lipid with an anionic 1,2-dioleoyl-*sn*-glycero-3-phosphate (DOPA) lipid did not yield a successful formulation (Methods). We injected C57BL/6 mice with either LNP^neut^ or LNP^cat^ carrying mRNA encoding aNLuc at a dose of 1 mg kg^–1^. Mice were dosed via the stomach or intravenously. Eighteen hours later, we isolated the same organs as in our previous screen and analyzed luminescence using an in vivo imaging system (IVIS). Both LNP^neut^ and LNP^cat^ demonstrated improved pancreas delivery and reduced targeting to other organs when administered via the stomach compared to intravenous administration (Figures 3c,d, S5, and S6). Gastric delivery of LNP^cat^ resulted in 27% greater total-fold flux change in the pancreas compared to LNP^neut^ delivered intragastrically and the greatest pancreas targeting of any combination tested. Intragastric administration of either LNP led to a reduction in off-target delivery compared to intravenous administration (Figure 3e–h).

**Figure 3:**
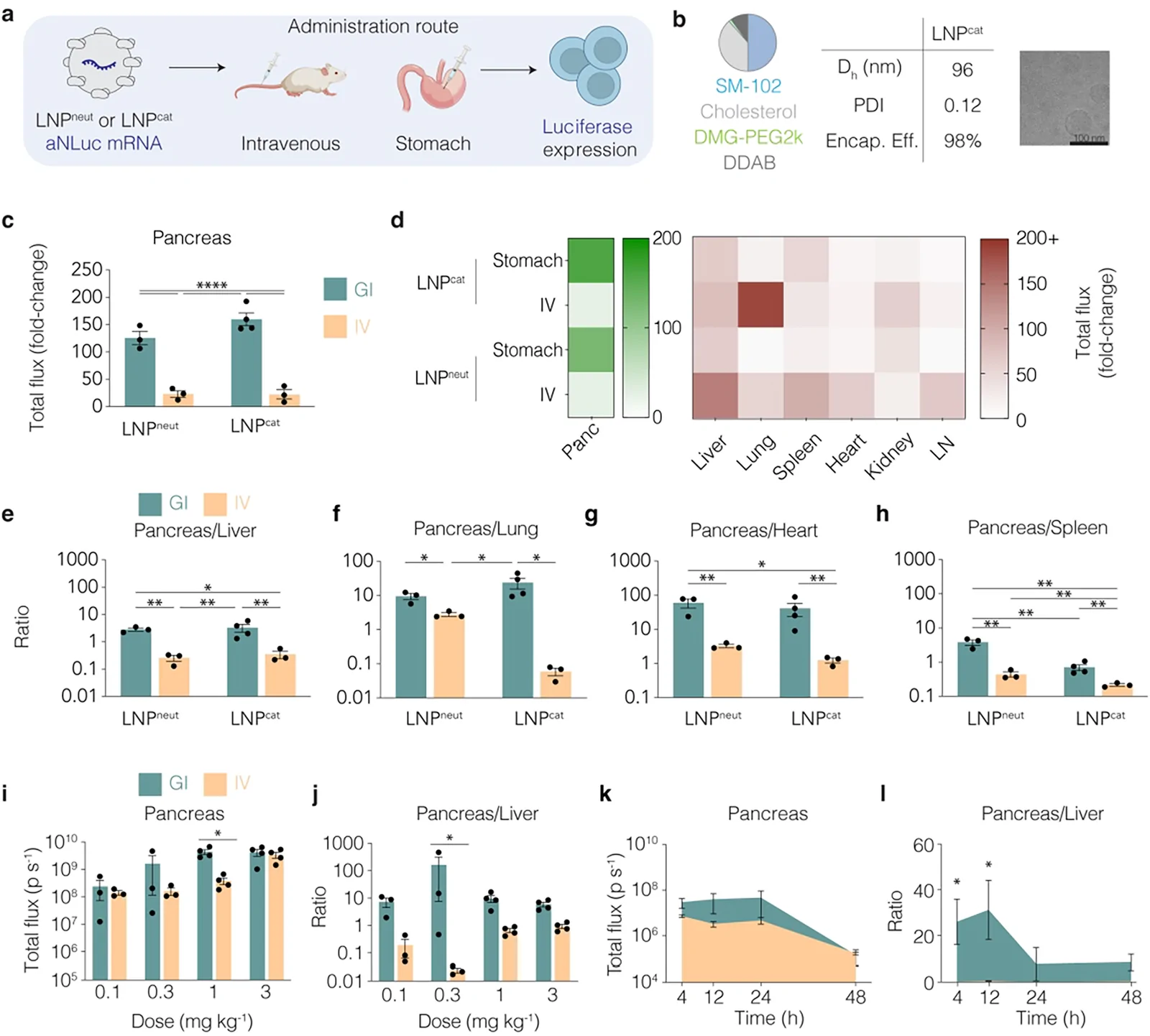
LNP^cat^ increases pancreas-targeting over LNP^neut^, with improved targeting effects seen when delivering via stomach compared to intravenous (IV) delivery. (a) aNLuc mRNA encapsulated in LNPcat was delivered via IV or stomach (GI) at 1 mg kg^–1^ and compared to phosphate-buffered saline dosed controls. IVIS imaging 18 h following dosing revealed luciferase expression. (b) Formulation details of LNP^cat^ and physicochemical characteristics: hydrodynamic diameter (*D*_h_), polydispersity index (PDI), encapsulation efficiency (Encap. Eff.), and representative cryogenic transmission electron microscope image. (c) Total flux (fold-change) of pancreas across IV and stomach administered mRNA with LNP^neut^ vs LNP^cat^ compared to 1× PBS group. (d) Heatmap showing total flux (fold change) between LNP^neut^ and LNP^cat^ across both IV and stomach administration routes to the pancreas (green), and off-target organs (e) liver, (f) lung, (g) heart, and (h) spleen (red). Additional off-target organs in Figure S6. (i, j) dose response of LNP^cat^ formulated with aNLuc mRNA 18 h postinjection showing (i) total flux quantification of pancreas and (j) pancreas/liver ratio. (k, l) Time-course of LNP^cat^ formulated with aNLuc mRNA delivered 0.3 mg kg^–1^, at 4 h, 12 h, 24 h, and 48 h. Quantified are (k) total flux of pancreas and (l) pancreas/liver ratios (*n* = 3–4 mice. Individual data points, mean ± SEM. Two-way ANOVA with a main effects-only model and Fisher’s LSD post-hoc test performed for LNP^neut^ vs LNP^cat^ studies. Multiple unpaired *t* tests with a single pooled standard deviation were performed for dose response and time course studies to compare GI vs IV groups across different doses or time points *p < 0.05; **p < 0.01; ****p < 0.0001.).

To investigate whether this trend of improved pancreas targeting via intragastric delivery over intravenous administration held across a range of doses and time points, we performed dose–response and time course studies. LNP^cat^ was formulated with aNLuc mRNA and administered via intravenous (IV) and gastric injection at 0.1, 0.3, 1.0, or 3.0 mg kg^–1^ in C57BL/6 mice. Eighteen hours after administration, we isolated the pancreas and liver and quantified delivery using an IVIS. Pancreas delivery increased with dose, and as expected, it saturated at a lower dose when performing gastric delivery relative to intravenous delivery (Figure 3i). Aiming for a maximal pancreas:liver ratio (Figure 3j), we chose the 0.3 mg kg^–1^ dose for our subsequent time course study. Specifically, we sacrificed mice at 4, 12, 24, and 48 h after LNP^cat^ administration. Total flux (p s^–1^) in the pancreas was stable for 24 h, then it decreased at the 48-h time point. This is consistent with previous time course studies.^35^ As expected from the previous findings, the pancreas-to-liver ratio was below 1 for IV administration throughout this time course, while it reached >20 for gastric administration (Figure 3k,l). Combined, these studies suggest that therapeutically relevant pancreas delivery can be achieved at lower doses and for longer time scales when delivered intragastrically compared to intravenously.

We then assessed the safety of both gastric and intravenous administration of the LNP^cat^. We administered a 3 mg kg^–1^ aNLuc mRNA dose to C57BL/6 mice. At 4 and 20 h after administration, we collected serum and quantified cytokines and liver damage enzymes. Serum interferon γ (IFNg), tumor necrosis factor α (TNFa), interleukin-6 (IL-6), and interleukin-12 subunit p40 (IL-12p40) levels from stomach-injected mice were within normal range and below those of the IV mice at both 4 and 20 h time points (Figure S7a–d). Notably, IFNg and IL-12p40 were elevated in IV mice 4 h after administration, but this resolved by 20 h. Liver enzymes alkaline phosphatase (ALP), aspartate aminotransferase (AST), alanine aminotransferase (ALT), and blood urea nitrogen (BUN) were all within normal levels in the stomach-injected groups, and all had lower values than the IV-injected groups. Only BUN was significantly greater than PBS-injected mice, but it was still within the range (Figure S7e–h). These results provide preliminary evidence that administering LNP^cat^ to the stomach is tolerated after a single 3 mg kg^–1^ dose.

### Intragastrically Administered GLP-1 mRNA as a Metabolic Modulator

We assessed the potential for intragastric mRNA dosing to carry out a metabolic therapeutic effect by delivering GLP-1 mRNA and subsequently conducting a glucose tolerance test (Figure 4a). We performed intragastric and subcutaneous delivery of GLP-1 mRNA formulated with LNP^cat^ at a dose of 0.3 mg kg^–1^. We compared these cohorts to those of mice dosed with phosphate-buffered saline. Eighteen hours after administration, and upon fasting the mice for 6 h, we observed that the gastric and subcutaneously dosed GLP-1 mRNA cohorts possessed a lower baseline fasting blood glucose level compared to the control group (Figure 4b). We then administered a 20% dextrose solution to both cohorts and measured blood glucose levels at 15, 30, 60, and 90 min time points. The GLP-1 mRNA-dosed groups possessed a lower blood glucose area under the curve across the entire study. We observed similar metabolic effects from the intragastrically and subcutaneously dosed drugs (Figure 4c,d).

**Figure 4:**
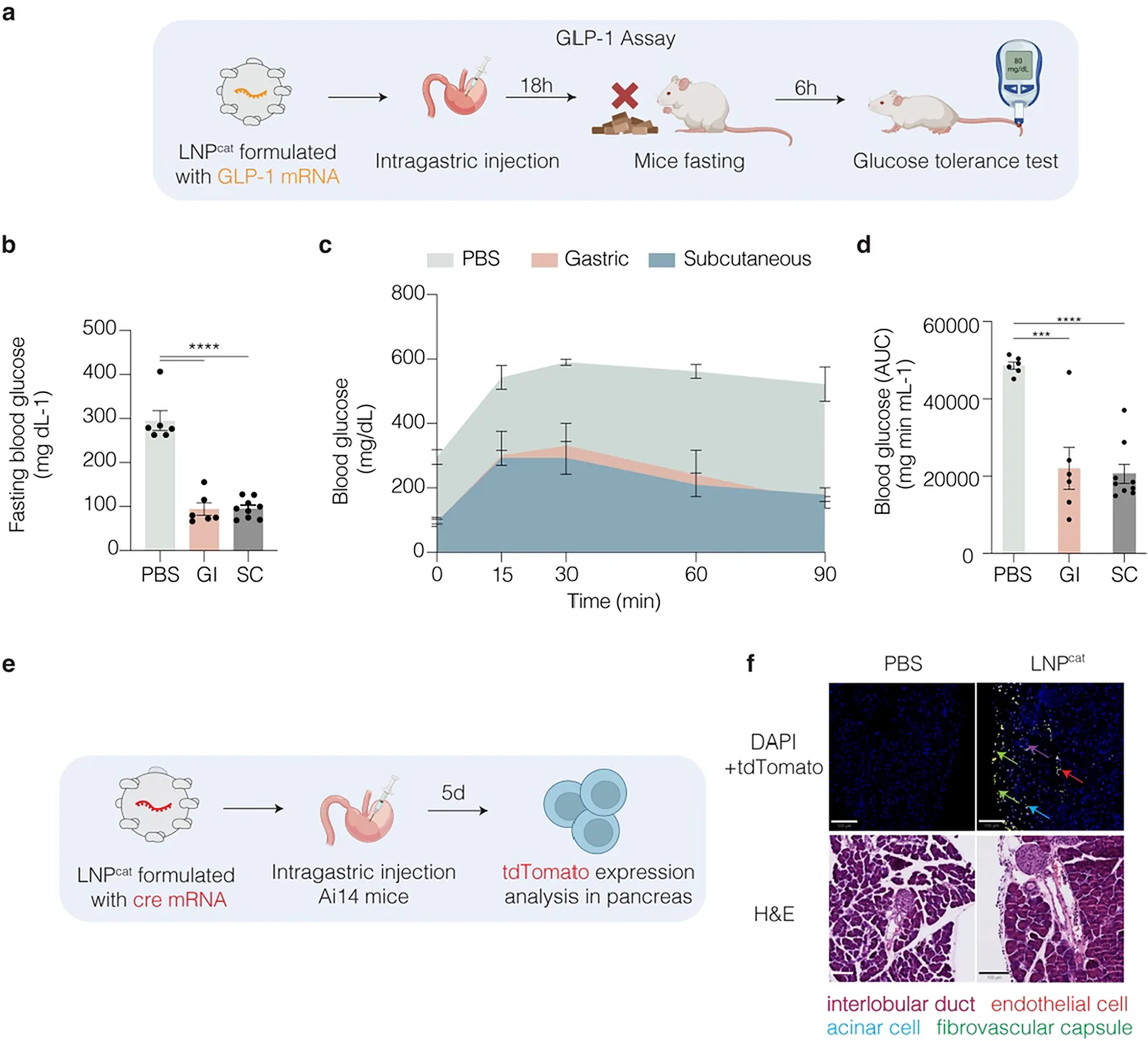
Gastric LNP administration can be used for metabolic mRNA therapies and targets multiple pancreatic cell types. (a) Timeline for intragastric (GI) administration of glucagon-like peptide-1 (GLP-1) mRNA LNP^cat^ or phosphate-buffered saline (PBS) to naïve C57BL/6 mice. (b) Fasting blood glucose 6 h after fasting in treated mice. SC, subcutaneous administration. (c) A glucose tolerance test was performed on mice 6 h after fasting by intragastric or subcutaneous administration of GLP-1 mRNA LNP^cat^ and subcutaneous administration of PBS. (d) Area under the curve (AUC) of glucose tolerance test for GLP-1 mRNA LNP^cat^ and PBS-treated mice. (e, f) Immunofluorescence (IF) targeting tdTomato following delivery of cre mRNA with LNP^cat^ in Ai14 mice shows targeted cell types in the pancreas 5 days after delivery, compared to PBS-treated mice. IF and hematoxylin and eosin (H&E) staining show targeting to multiple cell types. Scale bar = 100 μm. (*n* = 6–9 mice, (b, d) Individual data points. Mean ± SEM. One-Way ANOVA with Tukey’s correction. *p < 0.05; **p < 0.01; ***p < 0.001; ****p < 0.0001).

### Cellular Resolution of LNP^cat^ Delivery

One limitation of data sets generated with luciferase is the difficulty of determining which cell types are transfected. We therefore performed immunofluorescence (IF) to determine cell types targeted following GI and IV injections. We formulated LNP^cat^ with mRNA encoding Cre recombinase and administered this formulation to Ai14 mice via gastric and IV injections at a dose of 1 mg kg^–1^ (Figure 4e). Five days after administration, we collected pancreatic tissue for histology. We stained the tissue with an anti-tdTomato marker as well as with hematoxylin and eosin (Figures 4f and S8). Our histological analysis suggested that Cre-mediated genomic excision had occurred in pancreatic acinar cells, pancreatic endothelial cells, and cells within the interlobular duct and the fibrovascular capsule, whereas delivery to endothelial cells and islets occurred via IV administration.

## Conclusion

In this study, we systematically characterized the biodistributions of LNPs administered via five gastrointestinal delivery routes, including through submucosal buccal, gastric, small intestine, colonic, and rectal tissues. GI dosing strategies resulted in local GI uptake that exceeded that of IV dosing. Furthermore, gastric administration resulted in an order of magnitude increase in pancreas-specific delivery compared with IV administration, and buccal administration achieved notable lymphatic uptake. Using a Cre recombinase reporter system, we confirmed functional delivery across multiple pancreatic cell types, including acinar and endothelial cells. We also demonstrated the dual influences of nanoparticle chemistry and route of administration on organ tropism, with each targeting method enabling stacking targeting effects. Our findings suggest that drug-device combinations, such as robotic pills and endoscopes, can combine vehicle formulation strategies with mechanical tissue localization to maximize tissue-level targeting throughout the body. To fully realize this potential, additional studies in large animals and humans are needed to define the interplay among route of administration, particle chemistry, and biodistribution. Despite anatomical proximity, to our knowledge, no mechanism has been reported by which material moves locally between the stomach and pancreas via shared arterial or lymphatic routes. Protein corona composition is known to differ depending on the biochemical environment encountered at the injection site, which could contribute to the differences in tropism we observed across routes, but further mechanistic studies are needed.^42^ Our small-animal biodistribution mapping studies provide evidence that GI dosing can act as a method to reach organs that cannot be targeted parenterally while downregulating hepatic, lung, heart, and splenic expression.

In our studies targeting gastric administration, we evaluated the metabolic therapeutic effects of dosing GLP-1 mRNA. We successfully demonstrated more pronounced blood glucose regulation in our gastrically dosed cohorts compared with PBS controls. Additional studies in animals and humans will be required to understand the long-term pharmacokinetic and pharmacodynamic effects of GLP-1 mRNA delivery compared to the delivery of GLP-1 receptor agonists. In our studies targeting the lymphatic system, we evaluated buccal delivery of ovalbumin mRNA, successfully demonstrating comparable anti-OVA IgG titers to those of intramuscular administration at equivalent doses. Further vaccine experiments with larger cohorts, additional antigen types, and studies of mucosal immunity should also be explored to assess the potential advantages associated with GI vaccination routes.

GI dosing is especially relevant because of patient preference for oral delivery. Our findings point toward the development of gastric interfacing devices capable of delivering lipid nanoparticle payloads for the treatment of local and systemic pathologies. This approach provides a new administration route that promotes extrahepatic targeting with therapeutic potential, including the delivery of vaccines and metabolic control therapies.

## Methods

### mRNA Synthesis and Purification

aNLuc, ovalbumin, GLP-1, and Cre mRNA were synthesized by *in vitro* transcription (IVT). Gene including open reading frame (ORF) sequences were ordered as an IDT gene synthesis. The sequences of the GPI anchor and nanoluciferase have been reported in a previous study.^29^ The GLP-1 construct encoded the mature human GLP-1 peptide derived from the human proglucagon (GCG) transcript (NCBI RefSeq NM_002054.5) and was synthesized as a codon-optimized ORF containing an N-terminal secretion signal. The ovalbumin (OVA) construct encoded full-length chicken ovalbumin derived from the Gallus OVAL transcript (NCBI RefSeq NM_205152.3) and was synthesized as a codon-optimized ORF. Linear DNA templates for IVT were amplified by PCR using Phusion High-Fidelity PCR Master Mix with HF Buffer (New England Biolabs), forward primer, and a T120-containing reverse primer. The amplified PCR products were purified with the DNA Clean & Concentrator Kit (Zymo Research). IVT reactions were performed using the MEGAscript T7 Transcription Kit (Invitrogen) for 2 h at 37 °C. Cap 1 reagent (BOC Sciences) was used for cotranscriptional capping, and UTP was fully replaced with N1-methylpseudouridine-5′-triphosphate (N1-Me-pUTP, BOC Sciences). Following IVT, residual DNA was degraded by DNase I treatment for 15 min at 37 °C. The resulting IVT mRNA was purified by lithium chloride precipitation and analyzed by agarose gel electrophoresis to confirm integrity and purity.

### Lipid Nanoparticle Formulation

LNPs carrying aNLuc and Cre mRNA were formulated using the NanoAssemblr Ignite (Precision NanoSystems). SM-102 was purchased from Organix Inc., and cholesterol, poly­(ethylene glycol) (PEG) lipids, and helper lipids were purchased from Avanti Lipids. Aqueous (25 mM acetate) and organic (100% ethanol) phases were mixed at a flow rate ratio of 3:1 and a total flow rate of 12 mL min^–1^. An anionic LNP containing DOPA was not successfully formulated under the same conditions. Stabilizing this formulation would require modification of other core components, such as alternative lipid ratios and different starting buffers. For example, DOPA is not soluble in ethanol but is in a chloroform-containing solution. Making these changes would introduce confounding variables when isolating the effect of the surface charge. Therefore, we focused our charge-based comparisons on the LNP^neut^ and LNP^cat^.

### Nanoparticle Characterization

Nanoparticles were dialyzed using 100 kDa centrifugal filters into 10 mM Tris, then sterile purified through a 0.22 μm filter. The hydrodynamic diameter of the LNPs was measured using dynamic light scattering (DynaPro Plate Reader III, Wyatt), and the encapsulation efficiency was measured using the RiboGreen assay (Invitrogen).

### GI mRNA Delivery

All studies involving experimental animals were approved by the Georgia Institute of Technology Institutional Animal Care and Use Committee under protocol numbers A100590 and A100823. They were conducted in accordance with the Georgia Institute of Technology animal facility guidelines. C57BL/6J and Ai14 mice were purchased from The Jackson Laboratories. Sprague–Dawley rats were purchased from Charles River Laboratories.

### Characterization of Lipid Nanoparticles by Cryo-Transmission Electron Microscopy (cryo-TEM)

Samples (3 μL) of concentrated LNPs were placed on 300-mesh, copper grids, with a 20 nm-thick holey carbon substrate (1.2 μm hole diameter, 1.3 μm hole spacing; C-Flat). Grids were previously glow-discharged (negative charge) for 15 s using a GloQube Plus system (Quorum Tech, Laughton, U.K.). Using a Vitrobot Mark IV (Thermo Fisher Scientific, Hillsboro, OR), samples were blotted with filter paper for one second in a chamber maintained at room temperature and 100% humidity. They were then plunge-frozen into liquid ethane and stored in liquid nitrogen until cryo-TEM data acquisition.

Cryo-TEM grids under liquid nitrogen temperatures were loaded into a JEOL JEM-2200FS TEM instrument (JEOL, Japan) operating at 200 keV, using a Gatan 914 cryo-TEM sample holder. Micrographs were acquired using the Serial EM software^41^ with a Gatan K2 direct electron detector device (Direct Electron, San Diego, CA) under low-dose conditions and at nominal magnifications of either 3k× (overview images) or 40k×, yielding pixel sizes of 27.42 and 0.99 Å per pixel, respectively.

### Intragastric Stomach and Colonic Injections

6–8 week old mice were given one dose of ketoprofen 5 mg kg^–1^ subcutaneously (s.c.) during the surgical prep period. If they appeared to be in pain, ketoprofen was continued once a day for the next 2–3 days. Mice were anesthetized with isoflurane in an induction box and maintained on isoflurane for the duration of the procedure via a nose cone (2–3% isoflurane in oxygen for maintenance). The animal was placed in dorsal recumbency, and the abdomen was shaved from just cranial to the xiphoid process to just caudal to the umbilical area. The skin was aseptically prepared with alternating cycles of betadine and 70% ethyl alcohol. The animal was maintained on a heating pad (warm water circulating) and monitored for depth of anesthesia by quality of respiratory effort and response to toe pinch. A 1 cm incision was made on the ventral midline through both the skin and the peritoneal sac. Using atraumatic forceps and sterile cotton tip applicators, we stabilized the area of interest (stomach or colon—distal to the cecum). Using a 30-gauge needle, we injected the lipid nanoparticles suspended in sterile 10 mM Tris buffer into the subserosal side, targeting the submucosal space. We then delivered 50–100 μL of fluid based on the lipid nanoparticle concentration. To deliver this amount, we performed up to 10 injections per mouse (forming 10–15 μL boluses) in different locations of the tissue. For OVA cargo, dose escalation studies revealed that intragastric administration was poorly tolerated compared to buccal and intramuscular comparators. At 1 μg mRNA doses, 67% (*n* = 10) of the cohort died post mRNA administration compared to 35% of the cohort dying at a 0.1 μg dose (*n* = 10). This mortality was not observed with other mRNA cargos delivered intragastrically or other routes of administration, including a 0% mortality rate for buccal delivery of OVA cargo (*n* = 5) and nanoLuc cargo (*n* = 3). The abdominal wall was closed with 5–0 PDS or similar absorbable monofilament using a simple interrupted pattern. The skin was closed with a wound clip. Before recovery, mice were given sterile warm 0.9% NaCl s.c. at 20 mL kg^–1^. Mice were given buprenorphine sustained release at 1 mg kg^–1^ s.c. before recovery. Mice were recovered in a cage warmed by a heat lamp and monitored daily.

### Buccal and Rectal Injections

6–8 week old mice were anesthetized with isoflurane in an induction box and maintained on isoflurane for the duration of the procedure. Using a 30-gauge needle, we injected lipid nanoparticles (50–100 μL) submucosally into the buccal tissue underneath the cheek. Additionally, in other groups, we injected lipid nanoparticles by inserting a 30-gauge needle into the rectal lining and injecting 50–100 μL slowly. Mice were recovered in a cage warmed by a heat lamp and monitored daily.

### Intraduodenal Small Intestine Injections

Sprague–Dawley rats (200–300 g, Charles River) were given one dose of ketoprofen 5 mg kg^–1^ s.c. during the surgical prep period. Rats were anesthetized with isoflurane in an induction box and maintained on isoflurane for the duration of the procedure via a nose cone (2–3% isoflurane in oxygen for maintenance). The animal was placed in dorsal recumbency, and the abdomen was shaved from just cranial to the xiphoid process to just caudal to the umbilical area. The skin was aseptically prepared with alternating cycles of betadine and 70% ethyl alcohol. The animal was maintained on a heating pad (warm water circulating) and monitored for depth of anesthesia by quality of respiratory effort and response to toe pinch. A 1 cm incision was made on the ventral midline through both the skin and the peritoneal sac. Using atraumatic forceps and sterile cotton tip applicators, we stabilized the small intestine immediately distal to the pylorus. We then delivered 50–100 μL of fluid based on the lipid nanoparticle concentration. To deliver this amount, we performed up to 10 injections per mouse (forming 10–15 μL boluses) in different locations of the tissue. The abdominal wall was closed with 5–0 PDS or similar absorbable monofilament using a simple interrupted pattern. The skin was closed with a wound clip. Before recovery, rats were given sterile warm 0.9% NaCl s.c. at 20 mL kg^–1^. Rats were given buprenorphine sustained release at 1 mg kg^–1^ s.c. before recovery. Rats were recovered in a cage warmed by a heat lamp.

### Luciferase Assay

Eighteen hours postinjection, mice were sacrificed and organs (pancreas, spleen, kidney, liver, lung, heart, lymph node, buccal, small intestine, colon, stomach, and rectum) were extracted and incubated with 50 mg mL^–1^ Nano-Glo in PBS (Promega) for 20 min, shaking at 800 rpm (RPM) in a 24-well plate. After incubation, IVIS imaging (IVIS Spectrum CT, PerkinElmer) was performed. Tissues were placed in Nano-Glo Luciferase Assay substrate (Promega) in 24-well plates for 10 min, shaking at 100 rpm, and then measured. Bioluminescence was measured using the IVIS imaging system. Quantitative analysis was performed in Living Image software (PerkinElmer). Images were loaded as a group and brought onto the same scale. The same size regions of interest (ROIs) were applied to all organs for analysis. The percentage pancreas delivery in the organ screen was determined using the following equation for organs across the five routes of administration.

$${\mathrm{Total Flux Fold‐Change}}_{tissue_{X,i}}=\frac{{\mathrm{flux}}_{tissue_{X,i}}}{average\left({\mathrm{flux}}_{tissue_{X},untreated}\right)}$$

$$\%pancreas\;delivery=\frac{average\;flux_{pancreas}}{\sum_{X=1}^{all\;tissues}average\;flux_{tissue_{X}}}\times 100\%$$

### Ovalbumin-Specific IgG ELISA

OVA-specific IgG antibody titers were measured from serum collected as described above using an indirect ELISA. High-binding 96-well plates were coated with ovalbumin (Sigma-Aldrich, A2512) at 10 μg/mL in 1× coating buffer (Bio-Rad, BUF030) overnight at 4 °C. Plates were washed four times with PBS containing 0.2% Tween-20 (PBS-T) between all subsequent steps. Wells were blocked with 10% bovine serum albumin (BSA) in phosphate-buffered saline with Tween-20 for 2 h at room temperature. Serum samples were diluted 1:40 in ChonBlock Blocking/Sample Dilution ELISA Buffer (AMSBIO, 9068) and incubated for 2 h at room temperature. Goat anti-mouse IgG (H + L) Horseradish peroxidase (HRP)-conjugated secondary antibody (Invitrogen, Cat. #31430) diluted in ChonBlock Detection Antibody Dilution Buffer (AMSBIO, 90681) was then added and incubated for 1 h at room temperature. Plates were developed with the 3,3′,5,5′-tetramethylbenzidine (TMB) substrate (BioLegend, Cat. #421501) for 25 min and quenched with 2N sulfuric acid stop solution. Absorbance was read at 450 nm. A serial dilution of purified anti-OVA IgG standard (0.2–12.5 ng/mL; Chondrex, Cat. #3011) was included on each plate and processed identically alongside samples; antibody concentrations were interpolated from the resulting standard curve using log/log regression analysis.

### GLP-1 Assay

2–5-month-old mice were prepared as described previously for intragastric stomach injections. Mice were administered 6 μg of GLP-1 encoding mRNA encapsulated in LNP^cat^ intragastrically or subcutaneously. Eighteen hours post administration, GLP-1 mRNA-administered and PBS-administered mice were fasted for 6 h. A glucose tolerance test was subsequently performed. Blood glucose was measured via tail prick and an Arkray Glucocard Blood Glucose meter kit (Amazon, USA). After an initial measurement, mice were injected with 150 μL of 20% dextrose solution intraperitoneally. Measurements were taken at *t* = 15, 30, 60, and 90 min. The area under the curve was calculated with the trapezoidal rule.

### Tissue Sectioning and Imaging

Ai14 mice were injected via IV and stomach injections with PBS or 1 mg/kg of Cre mRNA encapsulated in LNP^cat^. Five days later, mice were sacrificed via isoflurane overdose and perfused with PBS and 10% Formalin. Pancreas and liver were extracted and submerged in 10% paraformaldehyde (PFA) for 48 h at 4 °C. After that, tissues were washed with 1× PBS 3 times for 15 min at room temperature and embedded in paraffin. Tissues were sliced to 50 μm using VT1000 S Vibratome (Leica). Sections were mounted on Superfrost Plus Microscope Slide (Thermo Fisher Scientific). Sections were generated for IF and H&E stains. To the slides stained with H&E, Vectashield Vibrance antifade mounting medium (Vector Laboratories) was added, then a coverslip was added. Imaging was performed using a spinning disk microscope (Nikon) at 10x zoom.

### Serum Cytokines and Blood Chemistry

Mouse sera were sent to IDEXX BioAnalytics and tested on the MILLIPLEX Mouse Cytokine/Chemokine Magnetic Bead Panel—Premixed 25 Plex (Millipore, cat. no. MCYTMAG70PMX25BK) according to the manufacturer’s protocol. Data were collected by xPONENT 4.3 (Luminex), and data analysis was completed using BELYSA 1.1.0 software. The data collected by the instrument software are expressed as median fluorescence intensity. Standard tox was performed by IDEXX to determine liver enzyme levels.

### Statistics

All statistics were performed in Excel or Prism (Graphpad, Version 10). Tests include One-Way ANOVA with Tukey’s correction and Two-Way ANOVA with Tukey’s correction. A minimum of three biological replicates were analyzed for quantification, except for PBS groups (minimum of two). All data are presented as the mean ± standard error of the mean.

## Supplementary Material





## Data Availability

All data associated with this study are present in the paper or the Supporting Information.

## Notes

Quantification of total flux (fold-change) (XLSX)
IVIS imaging data across gastrointestinal and intravenous administration routes; validation of injection integrity; dose–response and time-course studies; safety assessments including serum cytokine and liver enzyme analysis; and immunofluorescence imaging of pancreatic cell targeting (Figures S1–S8) (PDF)
