# Supplementary material for: Targeted Gastrointestinal Delivery of mRNA Lipid Nanoparticles Enables Systemic Vaccination and Glucagon-like Peptide‑1 Metabolic Therapy
Source: ACS Nano. 2026 Sep 28;20(40):26992–7001. doi: 10.1021/acsnano.5c18549 (PMC13671166; doi:10.1021/acsnano.5c18549)
Supplement: Supplementary file 1 [file nn5c18549_si_002.pdf]

Supporting information for the publication:

Targeted gastrointestinal delivery of mRNA lipid nanoparticles enables systemic vaccination and glucagon-like peptide-1 metabolic therapy

Abdulraouf M. Abbas[1,2,*], Ramy Ghanim[3,*], Sebastian Rudden[1], David Schultz[3], Bora Jang[1], Avraham Shakked[1], Hyejin Kim[1], James E. Dahlman[1,$], Alex Abramson[1,3,4,5,$]

[1]Wallace H. Coulter Department of Biomedical Engineering, Emory University School of Medicine and Georgia Institute of Technology, Atlanta, GA, 30332, USA

[2]Emory Global Diabetes Research Center of the Woodruff Health Sciences Center and Emory University, Atlanta, GA, 30322, USA

[3]School of Chemical and Biomolecular Engineering, Georgia Institute of Technology, Atlanta, GA, 30332, USA

[4]Wearable Intelligent Systems and Healthcare Center, Institute for Matters and Systems, Georgia Institute of Technology, Atlanta, GA, 30332, USA

[5]Division of Digestive Diseases, Emory University School of Medicine, Atlanta, GA, 30332, USA

*Equal Contribution

[$]Correspondence: aabramson6@gatech.edu, james.dahlman@emory.edu

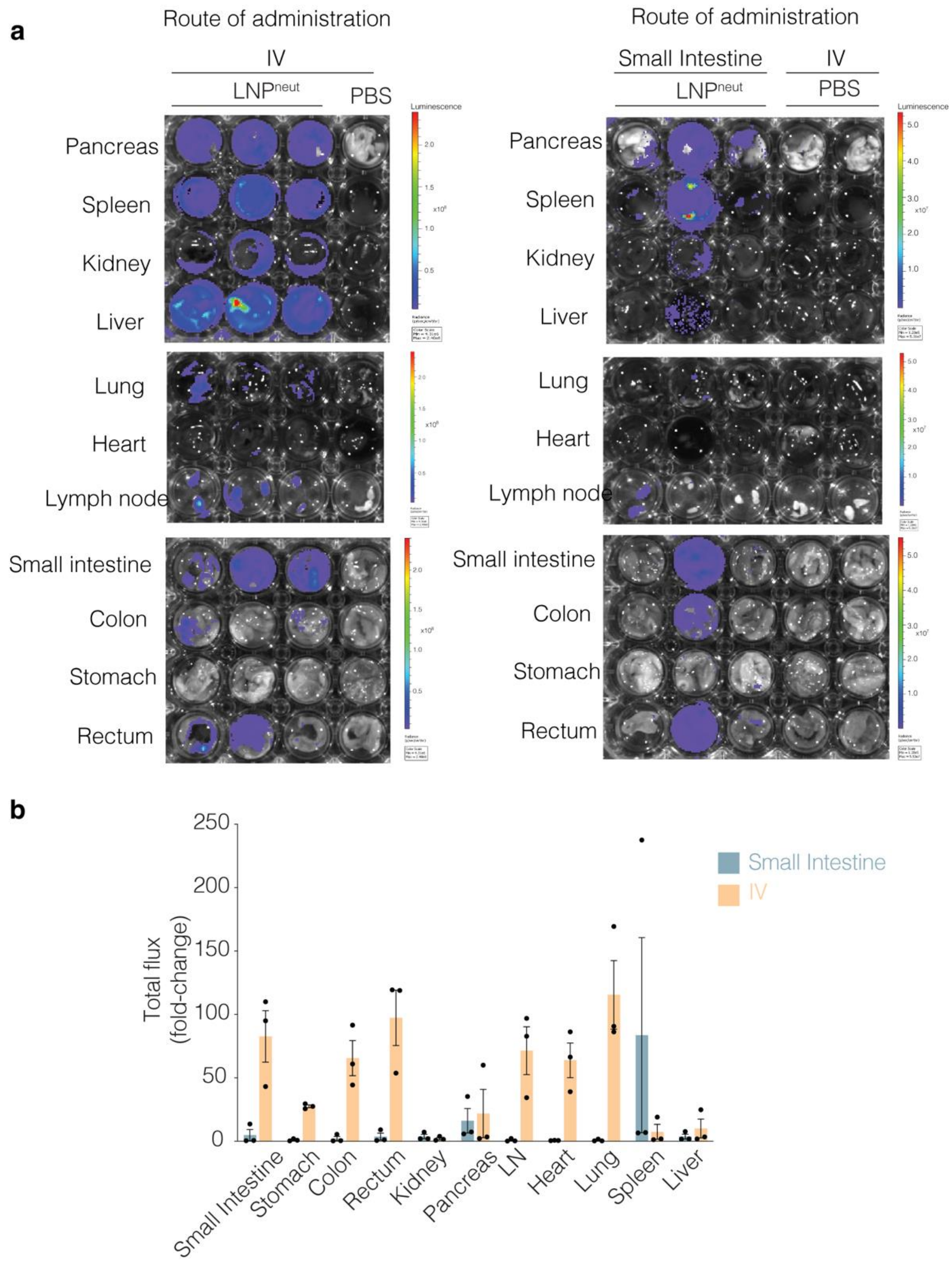

Figure S1. In vivo imaging system (IVIS) images after delivering aNLuc mRNA with LNP^{neut} via the small intestine in Sprague Dawley rats. aNLuc RNA was administered at a dose of 0.3 mg kg^{-1} through (a) intravenous (IV) and small intestine routes. (b) Total flux fold-change (n = 3, biologically independent animals, Mean ± SEM). Phosphate-buffered saline (PBS)-treated rats were used as negative controls and baseline for fold-change calculations. LN, lymph nodes.

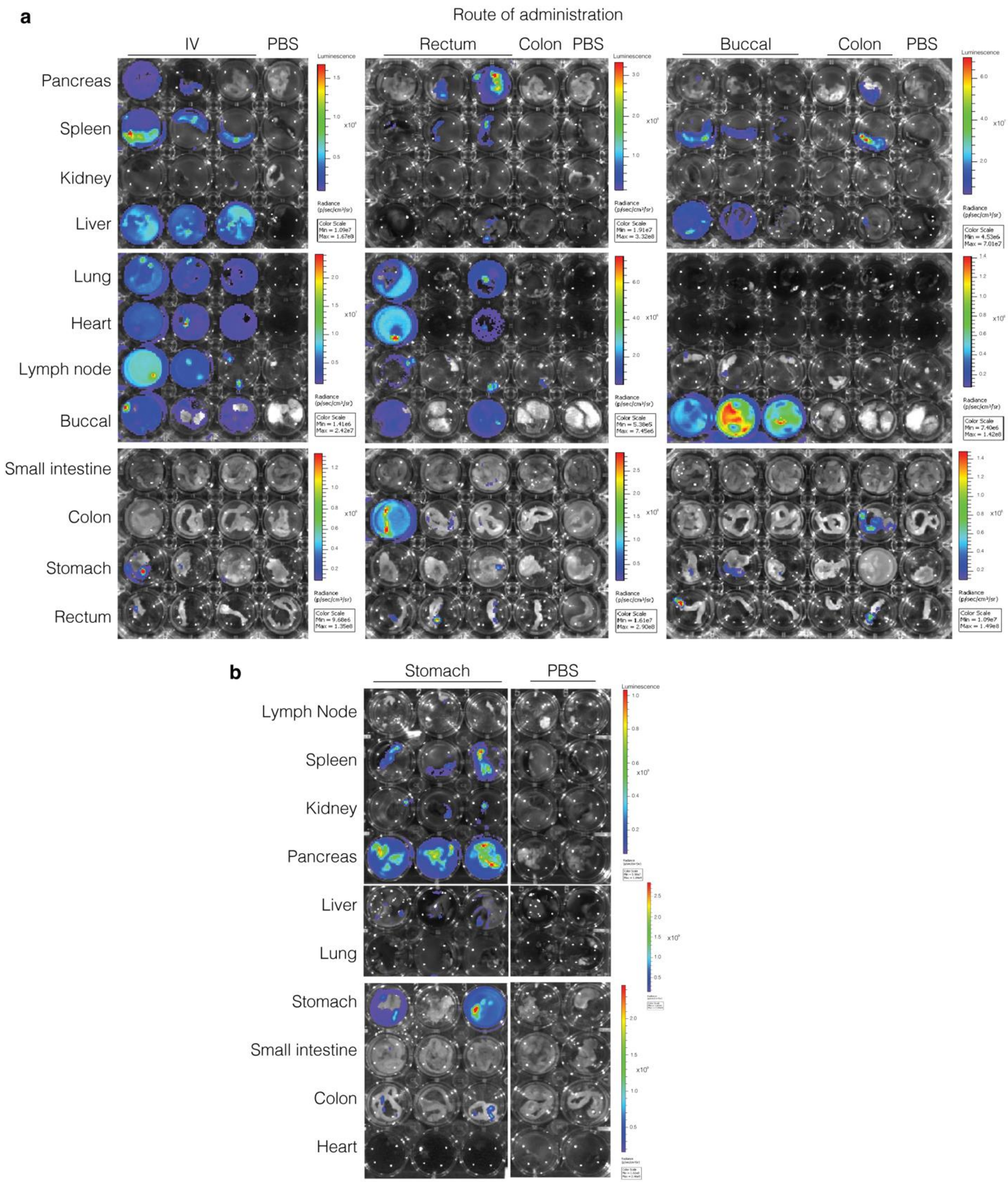

Figure S2. In vivo imaging system (IVIS) images of gastrointestinal organ delivery screen after delivering aNLuc mRNA with LNP^{neut}. aNLuc mRNA was administered at a dose of 1 mg kg^{-1} through (a) intravenous (IV), buccal, colon, rectum, and (b) stomach routes of administration. Luciferase expression was determined via IVIS imaging 18 h following

dosing. (n = 3, biologically independent animals). Phosphate-buffered saline (PBS)-treated mice were used as negative controls and baseline for fold-change calculations.

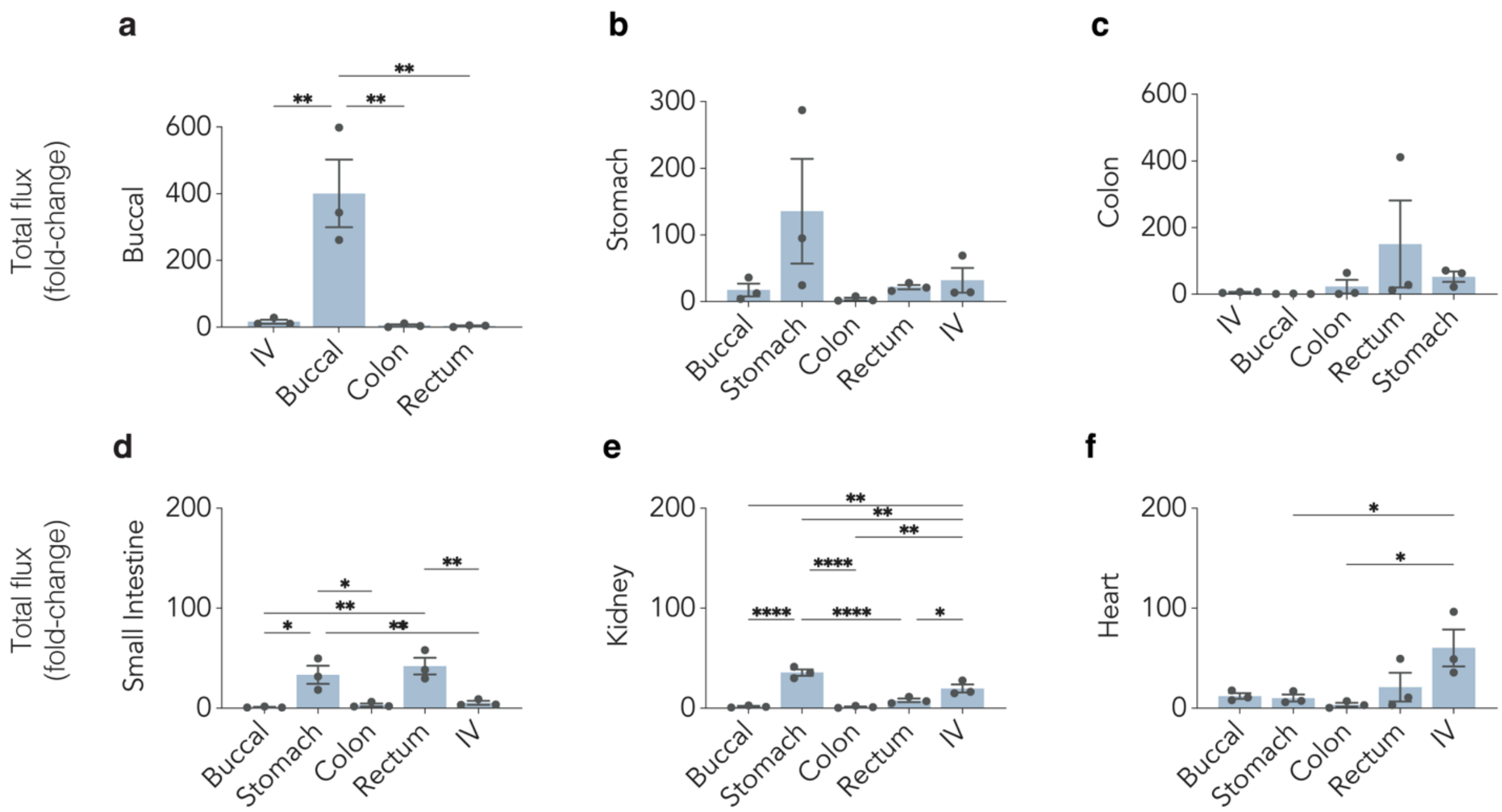

Figure S3. Total flux (fold-change) for additional organs in the gastrointestinal (GI) organ delivery screen. aNLuc RNA was administered at a dose of 1 mg kg^{-1} through multiple routes of administration: intravenously (IV), and to 4 locations across the GI tract - buccal, colon, rectum, and stomach submucosa tissue. Luciferase expression was determined via in vivo imaging system (IVIS) imaging at 18 h following dosing (n = 3, biologically independent animals). Total flux (fold-change) was quantified across tissues and administration routes: (a) buccal, (b) stomach, (c) colon, (d) small intestine, (e) kidney, and (f) heart, compared to phosphate-buffered saline (PBS)-treated controls. (n = 3-4 mice. Individual data points, Mean ± SEM. One-Way ANOVA with Tukey's correction. *p<0.05; **p<0.01; ****p<0.0001.)

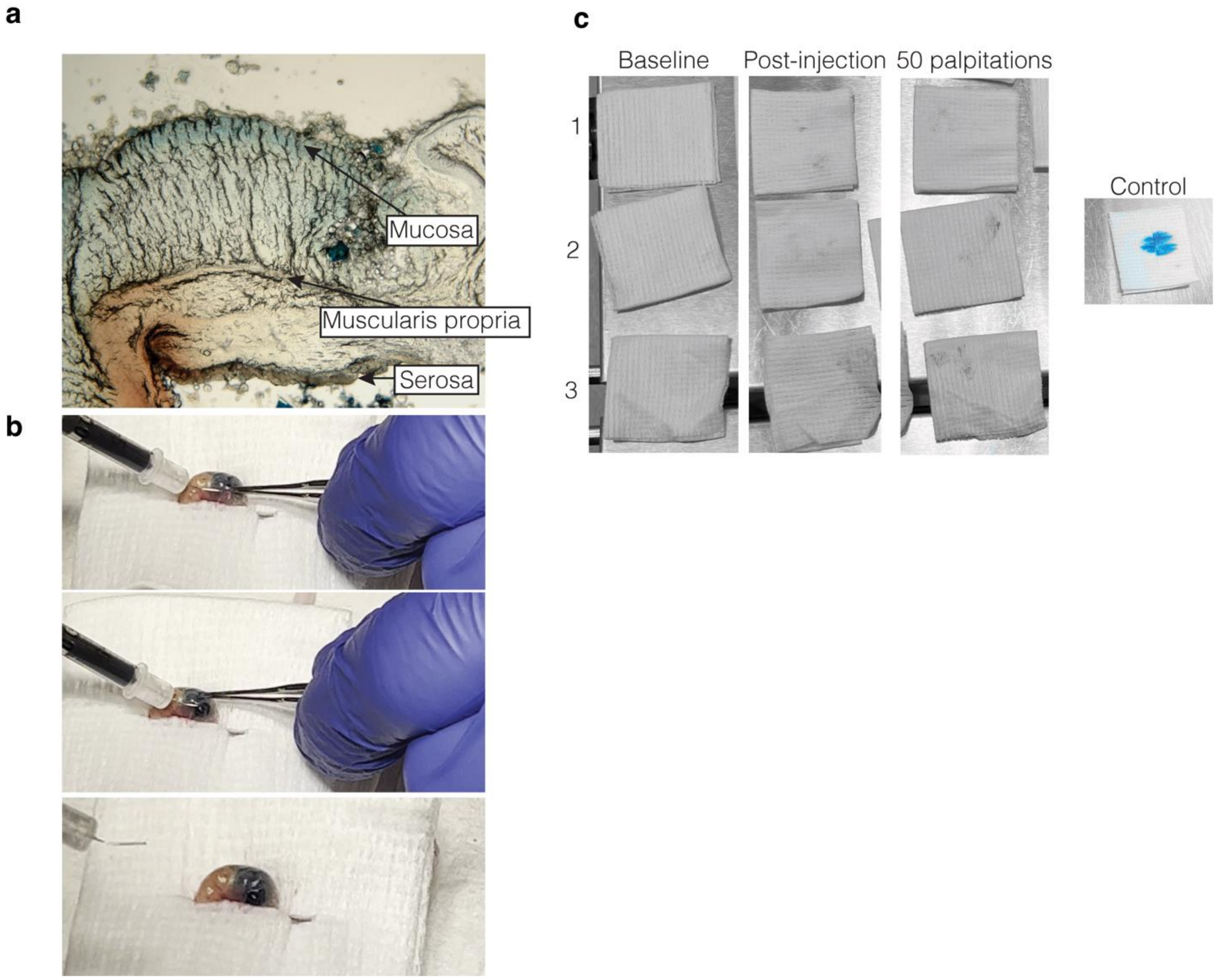

Figure S4. Testing gastric injection leakage with methylene blue to ensure no leakage. (a) Representative histology of 50 µm thick cross-section of murine stomach after subserosal injections targeting submucosa with 33G needle. (b) Representative images of injection technique. (c) Dry gauze applied to stomach: baseline-before injection, post-injection of 10 µL methylene blue, and after 50 palpitations of abdomen to agitate the affected area. The control gauze was sprayed onto with 10 µL of methylene blue.

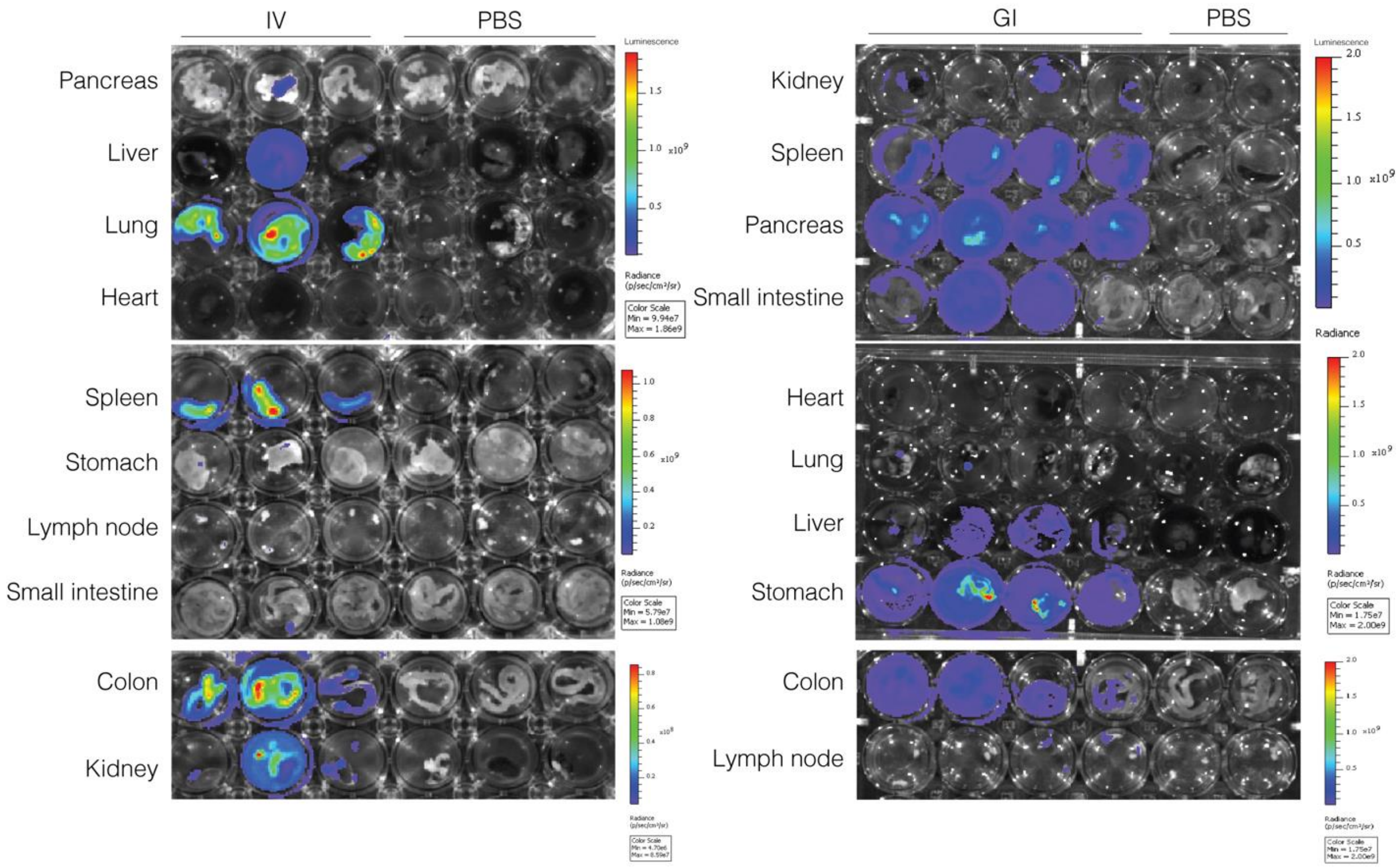

Figure S5. Images after administering aNLuc mRNA with LNP[cat] to stomach and intravenously. aNLuc mRNA encapsulated in LNP[cat] was delivered via intravenous (IV) or stomach (GI) delivery at a dose of 1 mg kg^{-1}. Luciferase expression was determined via in vivo imaging system (IVIS) imaging 18 h following dosing. Phosphate-buffered saline (PBS)-treated mice were used as negative controls and baseline for fold-change calculations.

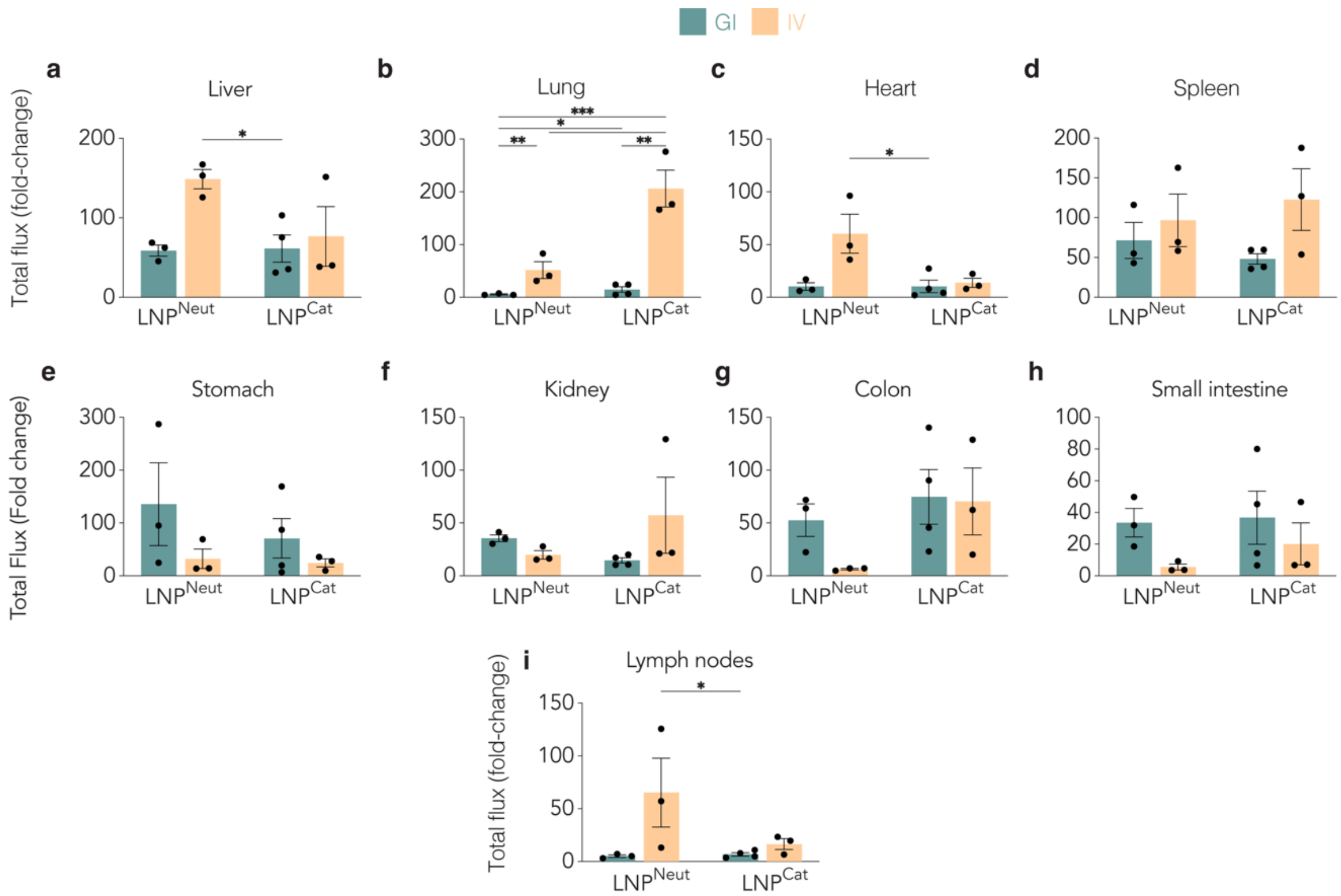

Figure S6. Total flux (fold change) for additional organs with aNLuc mRNA with LNP^{cat}. a-i) aNLuc mRNA encapsulated in LNP^{cat} was delivered via intravenous (IV) or stomach (GI) delivery at a dose of 1 mg kg^{-1}. Luciferase expression was determined via in vivo imaging system (IVIS) imaging at 18h following dosing. (n = 3 biologically independent animals). Quantification of total flux (fold-change) of (a) liver, (b) lung, (c) heart, (d) spleen, (e) stomach, (f) kidney, (g) colon, (h) small intestine, and (i) lymph nodes across IV and stomach administered mRNA with LNPneut vs LNPcat compared to phosphate-buffered saline (PBS)-treated group. (n = 3-4 mice. Individual data points, Mean ± SEM. Two-Way ANOVA with Tukey's correction. *p<0.05; **p<0.01; ****p<0.0001.)

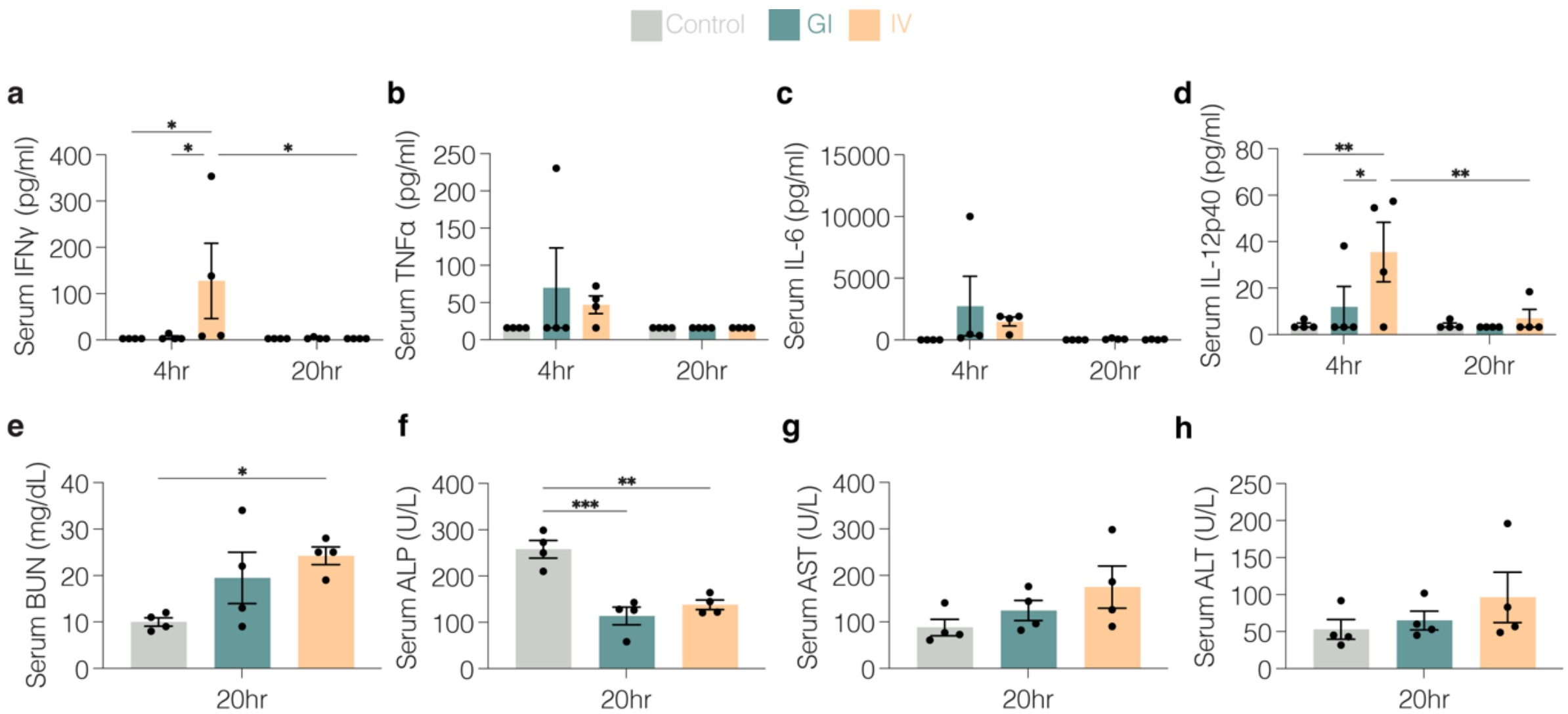

Figure S7. Safety profile following stomach (GI) and intravenous (IV) injections of 3 mg kg^{-1} aNLuc mRNA with LNP^{cat}. Mice were injected with 3 mg kg^{-1} mRNA encapsulated in LNP^{cat}, and serum was collected 4 hr and 20 hr post injection. Phosphate-buffered saline (PBS)-treated mice were used as negative controls (Control). a-d) Serum cytokines at 4 hr and 20 hr quantifying (a) Interferon-gamma (IFNγ), (b) Tumor necrosis factor alpha (TNFα), (c) interleukin-6 (IL-6), and (d) Interleukin-12 subunit p40 (IL-12p40). e-h) Blood chemistry was performed on serum to quantify liver enzymes at 20hr: (e) blood urea nitrogen (BUN), (f) alkaline phosphatase (ALP), (g) aspartate aminotransferase (AST), (h) alanine aminotransferase (ALT). (n = 3-4 mice. Individual data points, Mean ± SEM. One-Way ANOVA with Tukey's correction. *p<0.05; **p<0.01; ****p<0.0001.)

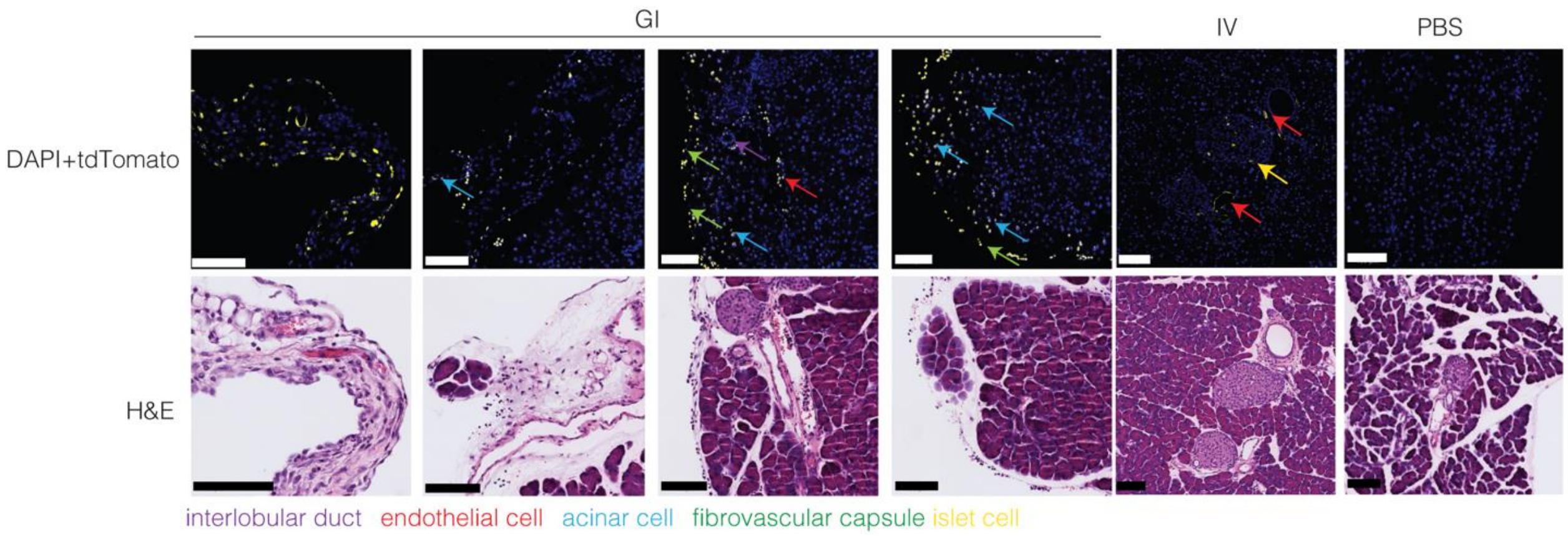

Figure S8. Immunofluorescence (IF) targeting tdTomato. Following delivery of Cre mRNA with LNP^{cat} in Ai14 mice via gastric (GI) and intravenous (IV) administration, IF shows targeted cell types in pancreas 5 days after delivery. IF and hematoxylin and eosin (H&E) staining shows targeting to multiple cell types as compared to a phosphate-buffered saline (PBS)-treated control. Scale bar = 100 μm.
